# Design, Synthesis and Biological Activity of 4,6-disubstituted Pyridin-2(1*H*)-ones as Novel Inhibitors of Soluble Epoxide Hydrolase

**DOI:** 10.22037/ijpr.2019.112047.13500

**Published:** 2019

**Authors:** Leila Hejazi, Elham Rezaee, Sayyed Abbas Tabatabai

**Affiliations:** *Department of Pharmaceutical Chemistry, School of Pharmacy, Shahid Beheshti University of Medical Sciences, Tehran, Iran.*

**Keywords:** Soluble epoxide hydrolase, Pyridinone, Docking, Inhibitor, Synthesis

## Abstract

Soluble epoxide hydrolase enzyme is a promising therapeutic target for hypertension, vascular inflammation, pain and some other risk factors of cardiovascular diseases. The most potent sEH inhibitors reported in the literature are urea-based ones which often have poor bioavailability. In this study, in a quest for finding potent inhibitors of soluble epoxide hydrolase, some 4,6-disubstituted pyridin-2(1*H*)-one derivatives were designed and synthesized. The designed compounds fit properly in the active site pocket of this enzyme in docking studies and have appropriate distances for effective hydrogen binding to important amino acids Tyr383, Tyr466, and Asp335. The results of biological evaluation of these compounds against soluble epoxide hydrolase enzyme indicate most compounds have acceptable inhibitory activity and compound **9c** is the most potent inhibitor with inhibitory activity of 86%.

## Introduction

Epoxide hydrolases (EHs) are a group of enzymes that convert epoxides from an oxidative metabolism of unsaturated endogenous and xenobiotic compounds to vicinal diols by the addition of water (1). Six sub-types of EH have been identified from which two types of mammalian EHs, microsomal epoxide hydrolase (mEH) and soluble epoxide hydrolase (sEH), have been intensely characterized. Soluble epoxide hydrolase (sEH), which is mostly expressed in the liver, kidneys, brain, endothelium and at lesser levels in other tissues, is the primary enzyme responsible for the conversion of epoxyeicosatrienoic acids (EETs) to their corresponding diols (2). EETs, but not their diols have potent biological activities: host defense, control of development, regulation of blood pressure, inflammation, and pain (1). Disruption of the sEH gene in male mice decreases blood pressure, and inhibition of sEH decreases blood pressure in several experimental hypertensive models (3). The sEH is more capable of degrading epoxy-fatty acids than the other xenobiotic epoxides *in-vivo* and hydrolyzes such epoxides 100-fold faster than other mammalian EHs (2-6). Because the effects of maintaining suitable EETs levels *in-vivo* has been largely beneficial, the inhibition of sEH has become a novel strategy to altering disease pathologies including cardiovascular diseases, and neurodegenerative disorders (7). Moreover, sEH inhibitors are a great deal of attention as well as selective cyclooxygenase 2 (COX-2) inhibitors as anti-inflammatory agents. The sEH inhibitors could be the first choice in inflammation disease due to low cardiovascular disease compare to selective COX-2 inhibitors (8). 

To date, several generations of sEH inhibitors have been developed which urea derivatives and carbamates are the latest developed classes of potent and selective sEH inhibitors (9). In our previous studies, we have developed diverse series of amide derivatives with significant sEH inhibitory activity and also identified an efficient pharmacophore model (10–12). The present investigation focused on the design and synthesis of pyridine-2(1*H*)-one ring linked to amide group as potential sEH inhibitors. Mechanistic studies of the designed compounds through *in-vitro* inhibition of sEH confirmed their inhibitory power for this enzyme and the results were correlated with *in-silico* data.

## Experimental

*Docking studies*

The high resolution crystal structure of sEH (PDB code: 3ANS) complexed with 4-cyano-*N*-[(1S,2R)-2-phenylcyclopropyl]benzamide was retrieved from RCSB Protein Data Bank. The enzyme and ligands are kept rigid and flexible respectively. Polar hydrogens and Kollman united atom partial charges were added to the individual protein atoms of enzyme. Each ligand structure was energy minimized under MM+ method in HyperChem 8 software and converted to pdbqt format file using AutoDockTools 4.0 version 1.5.6. (8, 13). 

Docking study was performed using AutoDock Vina version 1.1.2 (14). During pdbqt file preparation, polar hydrogen atoms and Kollman united atom partial charges were added to the protein atoms (sEH) (15). The grid box was set to 40 Å × 40 Å × 40 Å with a grid space value of 1 Å. The binding box was centered at x, y, and z coordinates of 25.8253, 24.3614, and 115.8739, respectively. All other docking parameters were set to their default values. Docking results were clustered with a root mean square deviation (RMSD) of 0.5 Å and visualized by Pymol software version 1.5.0.1.

*Chemistry*

All chemicals and reagents were purchased from Aldrich or Merck Company with a minimum purity of 97% and were used without further purification. The structures of the synthesized compounds were confirmed by IR, LC/MS, ^1^H NMR, and ^13 ^C NMR. IR spectra were recorded using KBr discs on a Perkin Elmer 843 IR. ^1^H NMR spectra were obtained with a Bruker Avance II (400 MHz) instrument using DMSO-d6 as solvent. They are reported as follows: chemical shifts δ in ppm (multiplicity, coupling constants *J *in Hz, number of protons, and assignment). Mass spectra were obtained on Agilent 6410 (QQQ) LC/MS system. Melting points were determined on an Electrothermal 9100 apparatus and are uncorrected.

*Synthesis of the Compounds*

*General Procedure for the Synthesis of the Compounds* ***1, 2a-h, 3***

Twelve milimole of substituded benzoyl chloride was diluted with anhydrous THF (10 mL) and added drop wise to a mixture of *p*-aminoacetophenone (10 mmol), Na_2_CO_3_ (10 mmol), and molecular sieves in anhydrous THF (10 mL) at room temperature and was stirred overnight. After reaction completion, the mixture was poured into ice-water and the precipitated solid was filtered off and recrystallized from ethanol. The same reagents and conditions were utilized to synthesize compounds **1** and **3** using succinic anhydride and phthalic anhydride respectively as acylating agents.

*4-((4-acetylphenyl)amino)-4-oxobutanoic acid* ***1***

White crystal, m.p. 233-235^ o^C, LC-MS: m/z 235.8 [M+H]^+^. 

*N-(4-acetylphenyl)benzamide* ***2a***

White powder, m.p. 204-206 °C, LC-MS: m/z 239.9 [M+H]^+^. 

*N-(4-acetylphenyl)-4-methylbenzamide* ***2b***

White powder, m.p. 226-227 °C, LC-MS: m/z 253.9 [M+H]^+^. 

*N-(4-acetylphenyl)-4-chlorobenzamide* ***2c***

White powder, m.p. 162-163 °C, LC-MS: m/z 273.9 [M+H]^+^. 

*N-(4-acetylphenyl)-4-fluorobenzamide* ***2d***

White powder, m.p. 112-114 °C, LC-MS: m/z 258.0 [M+H]^+^. 

*N-(4-acetylphenyl)-4-nitrobenzamide* ***2e***

Yellow crystals, m.p. 179-181 °C, LC-MS: m/z 284.9 [M+H]^+^. 

*N-(4-acetylphenyl)-3-chlorobenzamide* ***2f***

White powder, m.p. 101-102 °C, LC-MS: m/z 273.7 [M+H]^+^.

*N-(4-acetylphenyl)-3-methoxybenzamide* ***2g***

White powder, m.p. 88-90 °C, LC-MS: m/z 269.8 [M+H]^+^.

*N-(4-acetylphenyl)-2-fluorobenzamide* ***2h***

White powder, m.p. 89-91 °C, LC-MS: m/z 257.9 [M+H]^+^.

*2-((4-acetylphenyl)carbamoyl)benzoic acid* ***3***

White crystal, m.p. 237-238 °C, LC-MS: m/z 283.9 [M+H]^+^. 

*General Procedure for the Synthesis of the Compounds* ***4, 5a-q, 6***

Eight milimole of intermediate (**1, 2a-h, 3**) and 9 mmol arylaldehyde derivatives were dissolved in ethanol (30 mL), then NaOH 40% solution (6 mL) was added and stirred overnight at room temperature. Thereafter, the reaction mixture was poured into ice-water and the precipitate was filtered off and recrystallized from ethanol. 

*4-((4-cinnamoylphenyl)amino)-4-oxobutanoic acid* ***4***

White crystal, m.p. 192-195 °C, LC-MS: m/z 321.8 [M-1].

*N-(4-cinnamoylphenyl)benzamide* ***5a***

White powder, m.p. 178-180 °C, LC-MS: m/z 327.9 [M+H]^+^. 

*N-(4-cinnamoylphenyl)-4-methylbenzamide* ***5b***

White powder, m.p. 183-186 °C, LC-MS: m/z 342.0 [M+H]^+^. 

*4-chloro-N-(4-cinnamoylphenyl)benzamide* ***5c***

White powder, m.p. 185-186 °C, LC-MS: m/z 361.7 [M+H]^+^. 

*N-(4-cinnamoylphenyl)-4-fluorobenzamide* ***5d***

White powder, m.p. 158-160 °C, LC-MS: m/z 346.0 [M+H]^+^. 

*N-(4-cinnamoylphenyl)-4-nitrobenzamide* ***5e***

Yellow powder, m.p. 189-191 °C, LC-MS: m/z 372.7 [M+H]^+^. 

*3-chloro-N-(4-cinnamoylphenyl)benzamide* ***5f***

White powder, m.p.188-189 °C, LC-MS: m/z 361.8 [M+H]^+^. 

*N-(4-cinnamoylphenyl)-3-methoxybenzamide 5* ***g***

White powder, m.p. 204-207^ o^C, LC-MS: m/z 358.0 [M+H]^+^. 

*N-(4-cinnamoylphenyl)-2-fluorobenzamide 5* ***h***

White powder, m.p. 155-158^ o^C, LC-MS: m/z 345.8 [M+H]^+^. 

*N-(4-(3-(p-tolyl)acryloyl)phenyl)benzamide* ***5i***

White crystal, m.p. 160-161^ o^C, LC-MS: m/z 342.0 [M+H]^+^.

*N-(4-(3-(4-methoxyphenyl)acryloyl)phenyl)benzamide* ***5j***

White crystal, m.p. 197-199 °C, LC-MS: m/z 357.7 [M+H]^+^.

*N-(4-(3-(4-chlorophenyl)acryloyl)phenyl)benzamide* ***5k***

White crystal, m.p. 210-215 °C, LC-MS: m/z 361.7 [M+H]^+^.

*N-(4-(3-(4-fluorophenyl)acryloyl)phenyl)benzamide* ***5l***

White crystal, m.p. 155-158 °C, LC-MS: m/z 346.0 [M+H]^+^.

*N-(4-(3-(3-hydroxyphenyl)acryloyl)phenyl)benzamide* ***5m***

White crystal, m.p. 145-147 °C, LC-MS: m/z 344.0 [M+H]^+^.

*N-(4-(3-(3-methoxyphenyl)acryloyl)phenyl)benzamide* ***5n***

White crystal, m.p. 175-177 °C, LC-MS: m/z 357.8 [M+H]^+^.

*N-(4-(3-(2-methoxyphenyl)acryloyl)phenyl)benzamide* ***5o***

White crystal, m.p. 210-212 °C, LC-MS: m/z 357.7 [M+H]^+^.

*N-(4-(3-(2-chlorophenyl)acryloyl)phenyl)benzamide* ***5p***

White crystal, m.p. 207-208 °C, LC-MS: m/z 361.8 [M+H]^+^.

*N-(4-(3-(thiophen-2-yl)acryloyl)phenyl)benzamide* ***5q***

White crystal, m.p. 218-220 °C, LC-MS: m/z 333.9 [M+H]^+^.

*2-((4-cinnamoylphenyl)carbamoyl)benzoic acid* ***6***

White crystal, m.p. 145-148 °C, LC-MS: m/z 370.0 [M-1].

*Synthesis of 1-(2-amino-2-oxoethyl)pyridin-1-ium (* ***7*** *)*

Twenty-three gram (0.25 mol) of chloroacetamide and 25 g (0.31 mol) of pyridine were heated slowly and the clear solution dimmed at 85 °C and precipitated after 2 h. The pearl white solid was obtained by recrystallization from absolute ethanol. Pearl white crystal, m.p. 208 °C, LC-MS: m/z 137 [M].

*Synthesis of 4,6-diaryl-pyridin-2(1H)-one derivatives* ***(8, 9a-q, 10)***

To a mixture of 1,3-diaryl-2-propen-1-one (**4, 5a-5q, 6**) (2.0 mmol) and 1-(2-amino-2-oxoethyl)pyridin-1-ium chloride (**7**) (2.2 mmol) in methanol (10 mL), 1N aqueous solution of sodium hydroxide (2 mL) was added. The mixture was stirred at room temperature for 

24 h. The solvent was evaporated and solid precipitate was taken up in distilled water and then filtered. The product was purified by flash chromatography.

*4-oxo-4-((4-(6-oxo-4-phenyl-1,6-dihydropyridin-2-yl)phenyl)amino)butanoic acid* ***8***

Light yellow powder, m.p. 223-225 °C. ^1^H NMR (400 MHz, DMSO-*d*_6_) δ 11.66 (bs, 1H, COOH), 11.06 (bs, 1H, NH-pyridone), 10.24 (s, 1H, NH-amide), 7.86 (d, *J* = 8.4 Hz, 2H, H_3,5_-Phenylene), 7.81 (d, *J* = 8.4 Hz, 2H, H_2,6_-Phenylene), 7.70 (d, *J* = 8.8 Hz, 2H, H_2,6_-Phenyl), 7.51 (m, 3H, H_3,4,5_-Phenyl), 6.95 (s, 1H, H_5_-Pyridone), 6.60 (s, 1H, H_3_-Pyridone), 2.59 (d, *J* = 6 Hz, 2H, CH_2_CH_2_COOH), 2.54 (d, *J* = 7.2 Hz, 2H, CH_2_CH_2_COOH); ^13^C NMR (100 MHz, DMSO-*d*_6_) δ 174.41, 170.98, 164.15, 152.28, 141.11, 138.02, 129.84, 129.45 (2C), 128.00 (2C), 127.36 (2C), 119.14 (2C), 31.70, 29.36. IR (KBr) 3368, 1705, 1654, 1605 cm^-1^. LC-MS: m/z 360.8 [M-H]^-^.

*N-(4-(6-oxo-4-phenyl-1,6-dihydropyridin-2-yl)phenyl)benzamide* ***9a***

White powder, m.p. 265-267 °C. ^1^H NMR (400 MHz, DMSO-*d*_6_) δ 11.68 (bs, 1H, NH-pyridone), 10.46 (s, 1H, NH-amide), 7.99 (d, *J* = 6.8 Hz, 2H, H_2,6_-Benzamide), 7.96-7.92 (m, 4H, H_2_,_3,5,6_-Phenylene), 7.83 (d, *J* = 6.8 Hz, 2H, H_2,6_-Phenyl), 7.64-7.46 (m, 6H, H_3,4,5_-Phenyl, H_3,4,5_-Benzamide), 7.00 (s, 1H, H_5_-Pyridone), 6.63 (s, 1H, H_3_-Pyridone). ^13^C NMR (100 MHz, DMSO-*d*_6_) δ 166.23, 164.22, 152.32, 141.05, 138.02, 135.24, 132.22, 129.85, 129.45 (2C), 128.92 (2C), 128.20 (2C), 127.90 (2C), 127.38, 120.52. IR (KBr) 3297, 1641, 1605 cm^-1^. LC-MS: m/z 366.9 [M+H]^+^. 

*4-methyl-N-(4-(6-oxo-4-phenyl-1,6-dihydropyridin-2-yl)phenyl)benzamide* ***9b***

White powder, m.p. 286-289 °C. ^1^H NMR (400 MHz, DMSO-*d*_6_) δ 11.72 (bs, 1H, NH-pyridone), 10.37 (s, 1H, NH-amide), 7.92 (s, 4H, H_2_,_3,5,6_-Phenylene), 7.91 (d, *J* = 8 Hz, 2H, H_2,6_-Benzamide), 7.81 (d, *J* = 6.4 Hz, 2H, H_2,6_-Phenyl), 7.54-7.47 (m, 3H, H_3,4,5 _Phenyl), 7.35 (d, 2H, *J* = 8 Hz, H_3,5_-Benzamide), 7.00 (s, 1H, H_5_-Pyridone), 6.62 (s, 1H, H_3_-Pyridone), 251 (s, 3H, CH_3_); ^13^C NMR (100 MHz, DMSO-*d*_6_) δ 166.00, 164.18, 152.30, 142.30, 141.13, 138.01, 132.31, 129.86, 129.45 (4C), 128.24 (2C), 127.87, 127.38 (2C), 120.48, 21.51. IR (KBr) 3307, 1657, 1629 cm^-1^. LC-MS: m/z 381.1 [M+H]^+^. 

*4-chloro-N-(4-(6-oxo-4-phenyl-1,6-dihydropyridin-2-yl)phenyl)benzamide* ***9c***

White powder, m.p. 275-277 °C. ^1^H NMR (400 MHz, DMSO-*d*_6_) δ 11.70 (bs, 1H, NH-pyridone), 10.51 (s, 1H, NH-amide), 8.02 (d, *J* = 8.4 Hz, 2H, H_2,6_-Benzamide), 7.95-7.90 (m, 4H, H_2_,_3,5,6_-Phenylene), 7.83 (d, *J* = 6.4 Hz, 2H, H_2,6_-Phenyl), 7.64 (d, *J* = 8.4 Hz, 2H, H_3,5_-Benzamide), 7.54-7.47 (m, 3H, H_3,4,5-_Phenyl), 7.01 (s, 1H, H_5_-Pyridone), 6.63 (s, 1H, H_3_-Pyridone). ^13^C NMR (100 MHz, DMSO-*d*_6_) δ 165.08, 164.17, 152.29, 140.81, 138.00, 137.07, 133.90, 130.17 (2C), 129.86, 129.46 (2C), 129.01 (2C), 127.92, 127.38 (2C), 120.58. IR (KBr) 3297, 1654, 1619 cm^-1^. LC-MS: m/z 400.8 [M+H]^+^. 

*4-fluoro-N-(4-(6-oxo-4-phenyl-1,6-dihydropyridin-2-yl)phenyl)benzamide* ***9d***

White powder, m.p. 286-288 °C. ^1^H NMR (400 MHz, DMSO-*d*_6_) δ 11.80 (bs, 1H, NH-pyridone), 10.54 (s, 1H, NH-amide), 8.11-8.07 (dd, 8.8, 2 Hz, 2H, H_2,6_-Benzamide), 7.93 (s, 4H, Phenylene), 7.82 (d, *J* = 6.8 Hz, 2H, H_2,6_-Phenyl), 7.53-7.46 (m, 3H, H_3,4,5_-Phenyl), 7.39 (t, *J* = 8.8, 2H, H_3,5_-Benzamide)), 7.00 (s, 1H, H_5_-Pyridone), 6.64 (s, 1H, H_3_-Pyridone); ^13^C NMR (100 MHz, DMSO-*d*_6_) δ 165.08, 164.40, 163.40, 152.26, 140.95, 138.06, 131.03, 130.94, 129.82, 129.44 (2C), 127.87 (2C), 127.36 (2C), 120.56, 115.96, 115.74. IR (KBr) 3299, 1655, 1614 cm^-1^. LC-MS: m/z 384.9 [M+H]^+^. 

*4-nitro-N-(4-(6-oxo-4-phenyl-1,6-dihydropyridin-2-yl)phenyl)benzamide* ***9e***

Yellow crystal, m.p. 325-328 °C. ^1^H NMR (400 MHz, DMSO-*d*_6_) δ 11.73 (bs, 1H, NH-pyridone), 10.77 (s, 1H, NH-amide), 8.40 (d, *J* = 8.8 Hz, 2H, H_3,5_-Benzamide), 8.22 (d, *J* = 8.8 Hz, 2H, H_2,6_-Benzamide), 7.96 (d, *J* = 8.96 Hz, 2H, H_2,6_-Phenylene), 7.92 (d, *J* = 8.96 Hz, 2H, H_3,5_-Phenylene), 7.83 (d, *J* = 6.5 Hz, 2H, H_2,6_-Phenyl), 7.54-7.47 (m, 3H, H_3,4,5_-Phenyl), 7.02 (s, 1H, H_5_-Pyridone), 6.64 (s, 1H, H_3_-Pyridone); ^13^C NMR (100 MHz, DMSO-*d*_6_) δ 164.55, 164.18, 152.28, 149.72, 140.85, 140.51, 137.98, 129.87, 129.77 (2C), 129.46 (2C), 127.99 (2C), 127.38 (2C), 124.10, 120.69. IR (KBr) 3308, 1697, 1662 cm^-1^. LC-MS: m/z 412.1 [M+H]^+^. 

*3-chloro-N-(4-(6-oxo-4-phenyl-1,6-dihydropyridin-2-yl)phenyl)benzamide* ***9f***

White powder, m.p. 244-246 °C. ^1^H NMR (400 MHz, DMSO-*d*_6_) δ 11.70 (bs, 1H, NH-pyridone), 10.55 (s, 1H, NH-amide), 8.04 (s, 1H, H_2_-Benzamide), 7.96-7.90 (m, 5H, H_2_,_3,5,6_-Phenylene & H_6_-Benzamide), 7.83 (d, *J* = 6.8 Hz, 2H, H_2,6_-Phenyl), 7.70 (d, 1H, *J* = 8 Hz, H_4_-Benzamide), 7.60 (t, *J *= 8 Hz, 1H, H_5_-Benzamide), 7.52-7.50 (m, 3H, H_3,4,5_-Phenyl), 7.01 (s, 1H, H_5_-Pyridone), 6.63 (s, 1H, H_3_-Pyridone); ^13^C NMR (100 MHz, DMSO-*d*_6_) δ 164.72, 164.18, 162.29, 140.71, 138.00, 137.18, 133.73, 132.06, 130.96, 129.86, 129.46 (2C), 127.93 (4C), 127.38 (2C), 127.05, 120.61. IR (KBr) 3354, 1678, 1658 cm^-1^. LC-MS: m/z 400.9 [M+H]^+^. 

*3-methoxy-N-(4-(6-oxo-4-phenyl-1,6-dihydropyridin-2-yl)phenyl)benzamide* ***9g***

White powder, m.p. 232-234 °C. ^1^H NMR (400 MHz, DMSO-*d*_6_) δ 11.72 (bs, 1H, NH-pyridone), 10.44 (s, 1H, NH-amide), 7.94 (s, 4H, H_2_,_3,5,6_-Phenylene), 7.83 (d, *J* = 6.8 Hz, 2H, H_2,6_-Phenyl), 7.58 (d, *J* = 7.6 Hz, 1H, H_6_-Benzamide), 7.53-7.45 (m, 5H, H_3,4,5_-Phenyl & H_2,5_-Benzamide), 7.18 (d, *J* = 7.6, 1H, H_4_-Benzamide), 7.01 (s, 1H, H_5_-Pyridone), 6.64 (s, 1H, H_3_-Pyridone), 3.86 (s, 3H, OCH_3_); ^13^C NMR (100 MHz, DMSO-*d*_6_) δ 165.93, 164.22, 159.68, 152.34, 140.98, 138.01, 136.62, 130.10, 129.86, 129.45 (2C), 127.90 (2C), 127.38 (2C), 120.59 (2C), 120.40, 117.94, 113.46, 55.83. IR (KBr) 3270, 1636 cm^-1^. LC-MS: m/z 396.9 [M+H]^+^.

*2-fluoro-N-(4-(6-oxo-4-phenyl-1,6-dihydropyridin-2-yl)phenyl)benzamide* ***9h***

White powder, m.p**.** 266-268 °C. ^1^H NMR (400 MHz, DMSO-*d*_6_) δ 11.69 (bs, 1H, NH- pyridone), 10.64 (s, 1H, NH-amide), 7.93 (d, *J* = 8.4 Hz, 2H, H_2,6_ Phenylene), 7.85 (d, *J* = 8.4 Hz, 2H, H_3,5_-Phenylene), 7.84-7.82 (m, 2H, H_4,6_-Benzamide), 7.71 (t, *J* = 7.2 Hz, 1H, H_3_-Benzamide), 7.64-7.58 (m, 1H, H_5_-Benzamide), 7.54-7.47 (m, 3H, H_3,4,5_-Phenyl), 7.41-7.34 (m, 2H, H_2,6_-Phenyl), 7.00 (s, 1H, H_5_-Pyridone), 6.63 (s, 1H, H_3_-Pyridone); ^13^C NMR (100 MHz, DMSO-*d*_6_) δ 164.18, 163.44, 160.60, 152.30, 140.63, 138.00, 130.42, 130.39, 129.86, 129.46 (2C), 128.05 (2C), 127.38 (2C), 125.12, 125.09, 119.99, 116.81, 116.59. IR (KBr) 3205, 1656, 1626 cm^-1^. LC-MS: m/z 384.9 [M+H]^+^. 

*N-(4-(6-oxo-4-(p-methylphenyl)-1,6-dihydropyridin-2-yl)phenyl)benzamide* ***9i***

White powder, m.p. 287-290 °C. ^1^H NMR (400 MHz, DMSO-*d*_6_) δ 11.68 (bs, 1H, NH-pyridone), 10.46 (s, 1H, NH-amide), 7.99 (d, *J* = 7.2 Hz, 2H, H_2,6_-Benzamide), 7.95-7.90 (m, 4H, H_2_,_3,5,6_-Phenylene), 7.73 (d, *J* = 8 Hz, 2H, H_2,6_-Phenyl), 7.64-7.54 (m, 3H, H_3,4,5_-Benzamide), 7.32 (d, *J *= 8 Hz, 2H, H_3,5_-Phenyl), 6.97 (s, 1H, H_5_-Pyridone), 6.60 (s, 1H, H_3_-Pyridone), 2.37 (s, 3H, CH_3_); ^13^C NMR (100 MHz, DMSO-*d*_6_) δ 166.22, 164.21, 152.11, 141.01, 139.56, 135.23, 135.01, 132.23, 130.04 (2C), 128.93 (2C), 128.20 (2C), 127.87, 127.23, 120.50, 21.29. IR (KBr) 3473, 1657, 1629 cm^-1^. LC-MS: m/z 381.3 [M+H]^+^.

*N-(4-(6-oxo-4-(p-methoxyphenyl)-1,6-dihydropyridin-2-yl)phenyl)benzamide* ***9j***

White powder, m.p. 279-281 °C. ^1^H NMR (400 MHz, DMSO-*d*_6_) δ 11.63 (bs, 1H, NH-pyridone), 10.47 (s, 1H, NH-amide), 7.99 (d, *J* = 7.2 Hz, 2H, H_2,6_-Benzamide), 7.6-7.90 (m, 4H, H_2_,_3,5,6_-Phenylene), 7.80 (d, *J* = 8.8 Hz, 2H, H_2,6_-Phenyl), 7.64-7.54 (m, 3H, H_3,4,5_-Benzamide), 7.05 (d, *J* = 8.8 Hz, 2H, H_3,5_-Phenyl), 6.97 (s, 1H, H_5_-Pyridone), 6.59 (s, 1H, H_3_-Pyridone), 3.82 (s, 3H, OCH_3_); ^13^C NMR (100 MHz, DMSO-*d*_6_) δ 166.23, 164.25, 160.85, 151.74, 141.00, 135.24, 132.23, 129.99, 128.92 (2C), 128.75 (2C), 128.20 (2C), 127.87 (2C), 120.51, 114.81, 55.76. IR (KBr) 3065, 1625, 1622 cm^-1^. LC-MS: m/z 396.9 [M+H]^+^. 

*N-(4-(6-oxo-4-(p-chlorophenyl)-1,6-dihydropyridin-2-yl)phenyl)benzamide* ***9k***

Light yellow powder, Decompose at 310 °C. ^1^H NMR (400 MHz, DMSO-*d*_6_) δ 11.85 (bs, 1H, NH-pyridone), 10.46 (s, 1H, NH-amide), 7.99 (d, *J* = 6.8 Hz, 2H, H_2,6_-Benzamide), 7.93 (s, 4H, H_2_,_3,5,6_-Phenylene), 7.88 (d, *J* = 8.8 Hz, 2H, H_2,6_-Phenyl), 7.65-7.55(m, 5H, H_3,4,5_-Benzamide & H_3,5_-Phenyl), 7.01 (s, 1H, H_5_-Pyridone), 6.64 (s, 1H, H_3_-Pyridone); ^13^C NMR (100 MHz, DMSO-*d*_6_) δ 166.23, 164.21, 155.67, 150.94, 141.08, 136.78, 135.21, 132.26, 129.41 (2C), 129.28 (2C), 128.94 (2C), 128.20 (2C), 127.92, 120.49. IR (KBr) 3296, 1631, 1602 cm^-1^. LC-MS: m/z 400.9 [M+H]^+^. 

*N-(4-(6-oxo-4-(p-fluorophenyl)-1,6-dihydropyridin-2-yl)phenyl)benzamide* ***9l***

White powder, m.p. 278-281 °C. ^1^H NMR (400 MHz, DMSO-*d*_6_) δ 11.78 (bs, 1H, NH-pyridone), 10.46 (s, 1H, NH-amide), 7.98 (d, *J* = 6.8 Hz, 2H, H_2,6_-Benzamide), 7.93 (s, 4H, H_2_,_3,5,6_-Phenylene), 7.91-7.88 (m, 2H, H_2,6_-Phenyl), 7.64-7.54 (m, 3H, H_3,4,5_-Benzamide), 7.34 (t, *J* = 8.8, 2H, H_3,5_-Phenyl), 6.99 (s, 1H, H_5_-Pyridone), 6.62 (s, 1H, H_3_-Pyridone); ^13^C NMR (100 MHz, DMSO-*d*_6_) δ 166.22, 164.13, 162.17, 151.16, 141.06, 135.22, 134.42, 132.25, 129.73, 129.65, 128.93 (2C), 128.20 (2C), 127.91, 120.48, 116.41, 116.19. IR (KBr) 3254, 1740, 1643, 1614 cm^-1^. LC-MS: m/z 385.1 [M+H]^+^. 

*N-(4-(6-oxo-4-(m-hydroxyphenyl)-1,6-dihydropyridin-2-yl)phenyl)benzamide* ***9m***

White powder, m.p. 297-300 °C. ^1^H NMR (400 MHz, DMSO-*d*_6_) δ 11.75 (bs, 1H, NH-pyridone), 10.49 (s, 1H, NH-amide), 9.72(s, 1H, OH), 8.01 (d, *J* = 7.2 Hz, 2H, H_2,6_-Benzamide), 7.97 (d, *J* = 8.8 Hz, 2H, H_2,6_-Phenylene), 7.93 (d, *J* = 8.8 Hz, 2H, H_3,5_-Phenylene), 7.64-7.55 (m, 3H, H_3,4,5_-Benzamide), 7.33 (t, *J* = 8 Hz, 1H, H_5_-Phenyl), 7.24 (d, *J* = 8 Hz, 1H, H_4_-Phenyl), 7.17 (s, 1H, H_2_-Phenyl), 6.94 (s, 1H, H_5_-Pyridone), 6.92 (d, *J* = 8 Hz, 1H, H_6_-Phenyl), 6.57 (s, 1H, H_3_-Pyridone); ^13^C NMR (100 MHz, DMSO-*d*_6_) δ 166.24, 164.23, 158.32, 152.60, 148.19, 141.07, 139.46, 135.24, 132.22, 130.52, 129.73, 128.91(2C), 128.21 (2C), 127.90, 120.55, 118.09, 116.84, 114.04. IR (KBr) 3113, 1646, 1615 cm^-1^. LC-MS: m/z 383 [M+H]^+^. 

*N-(4-(6-oxo-4-(m-methoxyphenyl)-1,6-dihydropyridin-2-yl)phenyl)benzamide* ***9n***

White powder, m.p. 267-269 °C. ^1^H NMR (400 MHz, DMSO-*d*_6_) δ 11.71 (bs, 1H, NH-pyridone), 10.46 (s, 1H, NH-amide), 7.99 (d, *J* = 7.2 Hz, 2H, H_2,6_-Benzamide), 7.93 (s, 4H, H_2_,_3,5,6_-Phenylene), 7.59 (m, 3H, H_3,4,5_-Benzamide), 7.42 (t, 1H, *J* = 8 Hz, H_5_-Phenyl), 7.37 (d, 1H, *J* = 8 Hz, H_6_-Phenyl), 7.33 (s, 1H, H_2_-Phenyl), 7.06 (d, *J* = 8, 1H, H_4_-Phenyl), 6.99 (s, 1H, H_5_-Pyridone), 6.63 (s, 1H, H_3_-Pyridone), 3.86 (s, 3H, OCH_3_); ^13^C NMR (100 MHz, DMSO-*d*_6_) δ 166.22, 164.15, 160.19, 152.27, 141.03, 139.57, 135.23, 132.23, 130.53, 128.93 (2C), 128.20 (2C), 127.92 (2C), 120.49 (2C), 119.65, 115.59, 112.69, 55.75. IR (KBr) 3345, 1673, 1622 cm^-1^. LC-MS: m/z 396.9 

[M+H]^+^. 

*N-(4-(6-oxo-4-(o-methoxyphenyl)-1,6-dihydropyridin-2-yl)phenyl)benzamide* ***9o***

Light yellow powder, m.p. 244-246 °C. ^1^H NMR (400 MHz, DMSO-*d*_6_) δ 11.06 (bs, 1H, NH-pyridone), 10.45 (s, 1H, NH-amide), 7.98 (d, *J* = 7.2 Hz, 2H, H_2,6_-Benzamide), 7.92 (d, *J* = 8.8 Hz, 2H, H_2,6_-Phenylene), 7.84 (d, *J* = 8.8 Hz, 2H, H_3,5_-Phenylene), 7.59 (m, 3H, H_3,4,5_-Benzamide), 7.43 (m, 2H, H_4,6_-Phenyl), 7.15 (d, *J* = 8.4 Hz, 1H, H_3_-Phenyl), 7.05 (t, *J* = 7.4 Hz, 1H, H_5_-Phenyl), 6.78 (s, 1H, H_5_-Pyridone), 6.44 (s, 1H, H_3_-Pyridone), 3.83 (s, 3H, OCH_3_); ^13^C NMR (100 MHz, DMSO-*d*_6_) δ 166.22, 163.88, 156.75, 150.98, 140.89, 135.25, 132.22, 130.80, 130.27, 128.92 (2C), 128.20 (2C), 127.80, 127.74 (2C), 121.27, 120.58 (2C), 112.39, 56.11. IR (KBr) 3303, 1662, 1627 cm^-1^. LC-MS: m/z 396.8 [M+H]^+^. 

*N-(4-(6-oxo-4-(o-chlorophenyl)-1,6-dihydropyridin-2-yl)phenyl)benzamide* ***9p***

White powder, m.p. 252-254 °C. ^1^H NMR (400 MHz, DMSO-*d*_6_) δ 11.76 (bs, 1H, NH-pyridone), 10.46 (s, 1H, NH-amide), 7.98 (d, *J* = 7.2 Hz, 2H, H_2,6_-Benzamide), 7.92 (d, *J* = 8 Hz, 2H, H_2,6_-Phenylene), 7.87 (d, *J* = 8 Hz, 2H, H_3,5_-Phenylene), 7.61 (m, 2H, H_3,6_-Phenyl), 7.56 (m, 3H, H_3,4,5_-Benzamide), 7.48 (m, 2H, H_4,5_-Phenyl), 6.76 (s, 1H, H_5_-Pyridone), 6.38 (s, 1H, H_3_-Pyridone); ^13^C NMR (100 MHz, DMSO-*d*_6_) δ 166.23, 163.67, 155.49, 151.44, 141.08, 138.17, 135.22, 135.23, 131.31, 131.16, 130.74, 130.45, 129.65, 128.92 (2C), 128.20 (2C), 128.12, 127.81, 120.56. IR (KBr) 3373, 1654, 1643 cm^-1^. LC-MS: m/z 398.8 [M-H]^-^. 

*N-(4-(6-oxo-4-(thiophen-2-yl)-1,6-dihydropyridin-2-yl)phenyl)benzamide* ***9q***

Light yellow powder, Decompose at 350 °C. ^1^H NMR (400 MHz, DMSO-*d*_6_) δ 11.66 (bs, 1H, NH-pyridone), 10.48 (s, 1H, NH-amide), 7.99 (d, *J* = 7.2 Hz, 2H, H_2,6_-Benzamide), 7.94 (d, *J* = 8.8 Hz, 2H, H_2,6_-Phenylene), 7.88 (m, 3H, H_3,5_-Phenylene & H_3_-Thiophenyl), 7.75 (d, *J *= 4.8 Hz, 1H, H_5_-Thiophenyl), 7.58 (m, 3H, H_3,4,5_-Benzamide), 7.23 (t, *J* = 4.8 Hz, 1H, H_4_-Thiophenyl), 7.00 (s, 1H, H_5_-Pyridone), 6.44 (s, 1H, H_3_-Pyridone); ^13^C NMR (100 MHz, DMSO-*d*_6_) δ 166.24, 163.99, 145.43, 141.17, 140.83, 135.21, 132.25, 129.22, 129.02, 128.93 (2C), 128.21 (2C), 127.89 (2C), 127.80, 120.50 (2C). IR (KBr) 3330, 1717, 1674, 1654 cm^-1^. LC-MS: m/z 372.7 [M+H]^+^. 

*2-((4-(6-oxo-4-phenyl-1,6-dihydropyridin-2-yl)phenyl)carbamoyl)benzoic acid* ***10***

Light yellow powder, m.p. 205-207 °C. ^1^H NMR (400 MHz, DMSO-*d*_6_) δ 12.29 (bs, 1H, COOH), 11.29 (bs, 1H, NH-pyridone), 10.49 (s, 1H, NH-amide), 7.83-7.80 (m, 3H, H_3,4,6_-Benzamide), 7.74 (d, *J* = 8.4 Hz, 4H, H_2_,_3,5,6_-Phenylene), 7.60-7.58 (m, 1H, H_5_-Benzamide), 7.53-7.48 (m, 2H, H_2,6_-Phenyl), 7.44-7.40 (m, 3H, H_3,4,5_-Phenyl), 6.93 (s, 1H, H_5_-Pyridone), 6.57 (s, 1H, H_3_-Pyridone). ^13^C NMR (100 MHz, DMSO-*d*_6_) δ 168.14, 167.83, 164.12, 152.60, 141.46, 139.18, 137.91, 132.31, 130.32, 130.09, 130.02, 129.96, 129.49 (2C), 128.26, 128.04 (2C), 127.49, 127.41 (2C), 119.73. IR (KBr) 3058, 1655, 1623 cm^-1^. LC-MS: m/z 410.9 ([M+H]^+^).

*Evaluation of sEH inhibition*

The sEH inhibition was determined using Cayman fluorescence-based human soluble epoxide hydrolase assay kit (item number 10011671). The enzyme and the substrate (3-phenylcyano (6-methoxy-2-naphthalenyl) methyl ester-2-oxiraneacetic acid (PHOME)) were incubated at 25 °C with concentration of 50 nM of inhibitors for 15 min in 25 mM Bis-Tris/HCl buffer (200 µL; pH 7.0). 

The reference inhibitor for assay is 12-(3-adamantan-1-yl-ureido)dodecanoic acid (AUDA), one of the most effective inhibitors of sEH. The activity was determined by monitoring the appearance of 6-methoxy-2-naphthaldehyde by fluorescence detection with an excitation wavelength of 330 nm and an emission wavelength of 465 nm. All the synthesized 4,6-disubstituted pyridin-2(1*H*)-ones **8, 9a-q, 10**, and AUDA were dissolved in DMSO and tested in 50 nM concentration to the determination of the inhibitory activity.

**Table 1:** Inhibitory activity of the synthesized derivatives

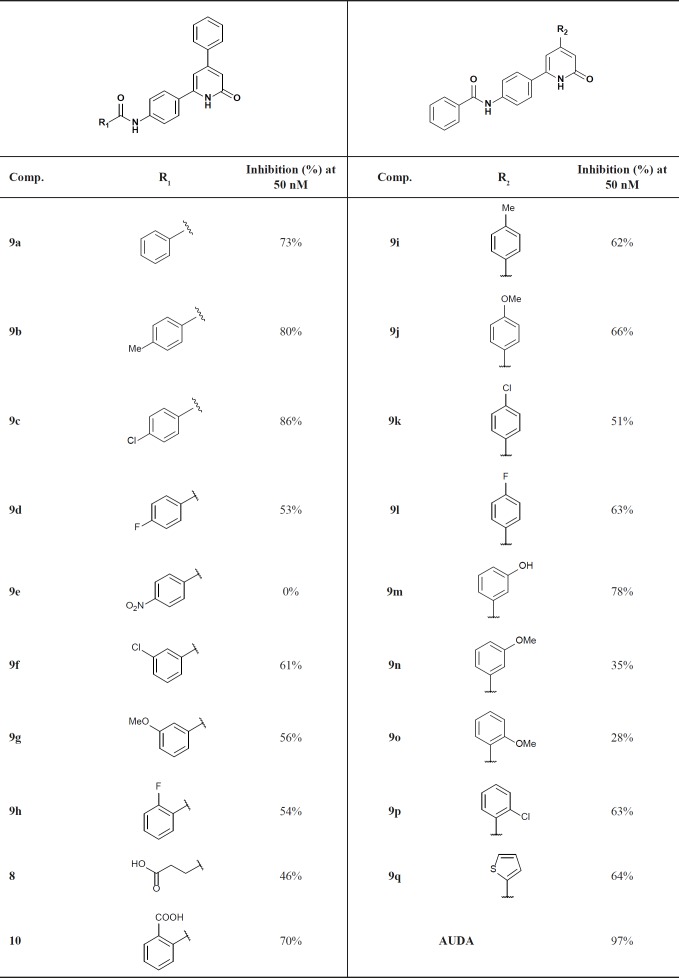

**Figure 1:**
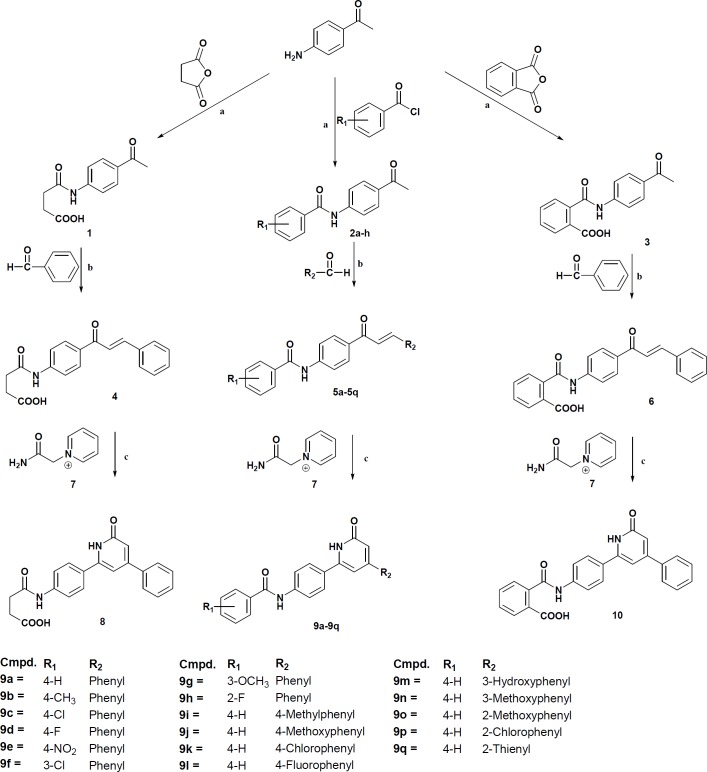
General synthetic methods. Reagent and conditions: (a) Na2CO3, anhydrous THF, r.t, 24 h; (b) NaOH, EtOH, r.t, 24 h; (c) NaOH.aq (1N), MeOH, r.t, 24 h.

**Figure 2:**
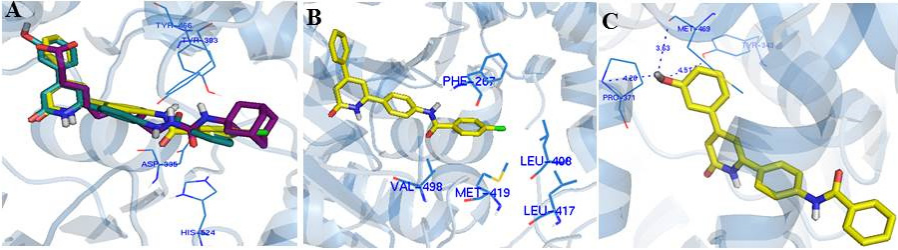
(A) Overlay of AUDA (magenta) with compounds 9c (yellow) and 9m (green) in the active site of she enzyme. (B) Placement of the lipophilic segment of 9c in the sEH hydrophobic cavity of the active site. (C) Hydrogen bonds between hydroxy group of 9m and Pro371, Met469 and Tyr 343.

## Results and Discussion

In this study, two series of pyridinone derivatives were synthesized according to Figure 1. The first series **8, 9a-9h, 10** includes pyridinone derivatives with different substituents in the benzamide moiety and the second series **9i-9q** involves pyridinone derivatives with various substituents in the phenyl ring. Intermediate compounds **4, 5a-q**, and **6** were prepared from the reaction of 4-aminoacetophenone with appropriate benzoyl chlorides, succinic and phthalic anhydrides following by reaction with proper aryl aldehydes (16). The final products **8**,** 9a-q** and** 10** were obtained from the reaction of 1,3-diaryl-2-propen-1-one derivatives with 1-(2-amino-2-oxoethyl)pyridin-1-ium (17). Molecular structures of the synthesized compounds were confirmed by IR, Mass, ^1^H NMR, and ^13^C NMR spectroscopies.

The assay protocols are described in the experimental section and the results are summarized in Table 1. Generally, in both groups of designed ligands, the amide group played the role of the primary pharmacophore and the pyridinone ring played the role of the secondary pharmacophore and effectively imitate the interactions of AUDA with the important amino acids in the sEH active-site such as Tyr383, Tyr466, Asp335, and His524 (Figure 2A). Moreover, the superimposing of compound** 9c, 9o**, and AUDA shows that the lipophilic segment of these ligands occupied exactly the same place in the enzyme as the lipophilic adamantyl group of AUDA (Figure 2A).

In the first group, derivatized from the benzamide fragment, the presence of lipophilic substitutions in *para*-position produced the highest inhibitory effect. Compound **9c** with an inhibitory activity of 86% has the strongest inhibitory effect in this category. The docking model shows that the lipophilic segment of **9c** is placed in the hydrophobic cavity of the active site, consisting of the lipophilic amino acids: Phe267, Leu408, Leu417, Met419, Val498 (Figure 2B). For this reason, the presence of lipophilic substituents in this region increases the potency through creating the hydrophobic bonds with the aforementioned amino acids. Interestingly, the placement of hydrophilic substituents in this position reduces the enzyme inhibition efficacy significantly since no inhibitory effect was observed for **9e** with 4-nitro substituent. Moreover, the presence of lipophilic substituents in the *meta*-position created an acceptable inhibitory effect, but the unsubstituted derivative is better. The presence of polar groups, *e.g.* flouro or carboxyl, in the *ortho*-position, compounds **9h** and **10**, maintained inhibitory activity, although the unsubstituted analogue is more active.

In the second group, different substituents with various sizes and electronic effects were placed in different positions of the phenyl ring. The results of the biological evaluation indicate that all compounds except the **9m** have a weaker inhibitory activity compared to their parent phenyl analogue **9a**. Compound **9m **with the hydroxyl group at the *meta*-position has an inhibitory activity of 78%. According to docking calculations, the hydroxyl substituent by making additional hydrogen bonds with Tyr343, Pro420, and backbone of Met469 increased the inhibitory effect of **9m** (Figure 2C). The ring bioisosterism was also used for more molecular modification in this series and phenyl ring was replaced by thiophene in compound **9q**. The physical properties of benzene and thiophene are very similar but thiophene is able to scavenge radicals and also shows better pharmacokinetic profile in developing new non-steroid anti-inflammatory agents (18,19). However, this change slightly decreased the inhibitory activity against sEH (**9q**: inhibitory activity = 64%). 

In summary, some new amide-based soluble epoxide hydrolase enzyme inhibitors with a pyridinone scaffold, as a novel secondary pharmacophore, were investigated. All of the novel synthesized compounds show acceptable inhibitory activity in comparison with AUDA. Compound **9c** was found to be the most potent inhibitor, with inhibitory activity value of 86% in 50 nM concentration. The docking study demonstrates that all compounds have affinity to the hydrolase catalytic pocket of sEH and fit properly. It seems that these structures could be a valuable lead scaffold to design and develop novel and potent sEH inhibitors.
